# The difference between trivial and scientific names: There were never any true cheetahs in North America

**DOI:** 10.1186/s13059-016-0943-y

**Published:** 2016-05-05

**Authors:** S. Faurby, L. Werdelin, J. C. Svenning

**Affiliations:** Section of Biology and Environmental Science, Department of Chemistry and Bioscience, Aalborg University, 9220 Aalborg, Øst Denmark; Department of Biogeography and Global Change, Museo Nacional de Ciencias Naturales, CSIC, Calle José Gutiérrez Abascal 2, 28006 Madrid, Spain; Swedish Museum of Natural History, Department of Palaeobiology, Box 50007, 10405 Stockholm, Sweden; Section for Ecoinformatics & Biodiversity, Department of Bioscience, Aarhus University, Ny Munkegade 114, 8000 Aarhus C, Denmark

## Abstract

Dobrynin et al. (Genome Biol 16:277, 2015) recently published the complete genome of the cheetah (*Acinonyx jubatus*) and provided an exhaustive set of analyses supporting the famously low genetic variation in the species, known for several decades. Their genetic analyses represent state-of-the-art and we do not criticize them. However, their interpretation of the results is inconsistent with current knowledge of cheetah evolution. Dobrynin et al. suggest that the causes of the two inferred bottlenecks at ∼ 100,000 and 10,000 years ago were immigration by cheetahs from North America and end-Pleistocene megafauna extinction, respectively, but the first explanation is impossible and the second implausible.

## Correspondence

Dobrynin et al. [1] appear to take the common name of the extinct North American cat *Miracinonyx*, which often is called the “American cheetah” to mean that the species is a close relative of the true cheetah, even though they correctly cite that ancient DNA shows *Miracinonyx* spp. nested within the genus *Puma* [2]. This is based on a single mitochondrial marker and could represent mitochondrial capture masking a true relationship between *Miracinonyx* and *Acinonyx*, but this molecular result was taken by its authors as support of prior morphological analysis showing that many of the specifically “cheetah-like” adaptations of *Miracinonyx* are convergences due to similar life styles [3]. No fossils of *Acinonyx* are known from North America, despite the extensive fossil record from the continent, while no fossils of *Miracinonyx* are known outside North America. Finally, both *Acinonyx* and *Miracinonyx* originated more than two million years ago [3] so that even if *Acinonyx* and *Miracinonyx* were sister genera, *Miracinonyx* would still not affect the population dynamics of Late Pleistocene *Acinonyx*. Thus, North American species are irrelevant to explaining the results obtained by Dobrynin et al.

The interpretation by Dobrynin et al. for the second bottleneck, though not impossible, is also unlikely. The authors apparently suppose that megafaunal extinction occurred 10–12,000 years BP globally but all the examples of extinctions they mention are North American, a continent which, as we already discussed, never had any true cheetahs. Extinctions in Africa, which are much more relevant to cheetahs, are more prolonged and go back to the early Pleistocene [4]. We note also that the natural range of *Acinonyx* includes some of the areas of the world with the smallest Late Pleistocene megafauna extinctions [5]. Hence, tying the inferred population bottleneck to megafauna extinction seems problematic in an African context.

Dobrynin et al. used two methods to infer population history: DaDi software and the pairwise sequentially Markovian coalescent (PSMC) model. Only DaDi inferred two bottlenecks, whereas PSMC indicated continuous population decline. They suggest that the differences are because the power of PSMC is inadequate to infer the bottlenecks but they do not appear to have tested the preferred model of PSMC (of a gradual consistent decline) in DaDi, making it difficult to compare the two methods. We also caution about the precision of the estimated dates. Johnson et al. [6] estimate the divergence time between *Felis* and *Acinonyx* to 6.7 million years ago, which is close to the estimate of 7 million years ago used by Dobrynin et al., but Johnson et al [6] give a confidence interval of 5.3–9.2 million years. Generation times are equally tricky. Dobrynin et al. use 3 years, which is intermediate between the age of first reproduction of 2 years and the average generation interval of 5.3 years estimated by Kelly [7]. Combining these uncertainties, we cannot be sure if the inferred events are actually twice or half as old as reported.

We think that the simplest explanation of the patterns may be a gradual decline in *Acinonyx* as inferred by the PSMC algorithm, potentially with sharper declines in some periods as suggested by the DaDi analysis. *Homo sapiens* first appeared ~200,000 years ago [8] and significant advances in the culture and technology of this species took place in the subsequent 100,000 years, both in South and central Africa [9, 10] (no data are available for eastern Africa). This may suggest a role for *H. sapiens* in the population dynamics of cheetahs, most likely as a consequence of interference competition. Such a process has been suggested for early Pleistocene large carnivore extinctions in Africa, potentially as a consequence of interactions with early *Homo* [4].

In conclusion, we find that the hypotheses of Dobrynin et al. to explain the inferred population bottlenecks of cheetahs lack support from either molecular systematics, paleontology, or the history of the African mammal fauna. We suggest a possible alternative scenario based on the events in Africa during the past 200,000 years, a scenario that requires further testing but that is plausible given available knowledge.
